# Application of Collocated GPS and Seismic Sensors to Earthquake Monitoring and Early Warning

**DOI:** 10.3390/s131114261

**Published:** 2013-10-24

**Authors:** Xingxing Li, Xiaohong Zhang, Bofeng Guo

**Affiliations:** 1 School of Geodesy and Geomatics, Wuhan University, 129 Luoyu Road, Wuhan, Hubei 430079, China; E-Mails: lxlq109121@gmail.com (X.L.); guobofeng@whu.edu.cn (B.G.); 2 German Research Centre for Geosciences (GFZ), Telegrafenberg, Potsdam 14473, Germany; E-Mail: lixin@gfz-potsdam.de

**Keywords:** real-time high-rate GPS, seismic sensor, integrated displacements, earthquake monitoring, earthquake early warning

## Abstract

We explore the use of collocated GPS and seismic sensors for earthquake monitoring and early warning. The GPS and seismic data collected during the 2011 Tohoku-Oki (Japan) and the 2010 El Mayor-Cucapah (Mexico) earthquakes are analyzed by using a tightly-coupled integration. The performance of the integrated results is validated by both time and frequency domain analysis. We detect the P-wave arrival and observe small-scale features of the movement from the integrated results and locate the epicenter. Meanwhile, permanent offsets are extracted from the integrated displacements highly accurately and used for reliable fault slip inversion and magnitude estimation.

## 1. Introduction

Earthquake early warning, which is the rapid detection of an ongoing earthquake, prediction of the expected ground shaking based on information extracted from the early arriving P-waves, and transmission of a useful warning prior to the onset of damaging ground shaking, is considered to be an effective, pragmatic, and viable tool for the earthquake emergency response and seismic hazard mitigation [1,2]. In the standard approaches, earthquake early warning systems have historically been based on seismic sensors. Such systems have been implemented in seismically active seismic regions, e.g., Japan, Mexico and California [3–5].

Earthquake-induced coseismic displacement is the essential information required for rapid source and rupture inversion. However, due to the rotation, tilt, drift and saturation problem of seismic instruments (strong motion sensors and broadband seismometer) and imprecision in the numerical integration process, the integrated displacements are not reliable in real-time [6]. Recent advances in the performance of real-time high-rate GPS, estimates of permanent displacement directly, mean that its use can potentially be complementary to the seismic-based methodologies for earthquake early warning [1,7–10]. The main weaknesses of current GPS measurements are the lower sampling rates (1∼50 Hz) and the larger high-frequency noise contribution [11], and so the GPS-derived dynamic motions are not accurate enough to identify the first arrival wave (P-wave). While strong motion sensors are able to sample at very high rates (e.g., 200 Hz) and perform very well in the high-frequency range as it is much more sensitive to ground motions than GPS receiver, especially in the vertical direction. The complementary nature of GPS and seismic sensors for station displacement estimation and P-wave detection is well recognized and the integrated processing of the two dataset is a hot topic in GPS seismology for obtaining more accurate and reliable displacements and P-wave arrival time [12,13].

Several loosely-integrated approaches have been proposed to fuse accelerometer with collocated GPS displacement data [14–18]. As the GPS coordinates are already estimated prior to integration with the accelerometer, the precise dynamic information provided by accelerometers cannot be used to enhance the GPS-only solutions in these integration algorithms. In order to combine all the advantages of both sensors, Li *et al.* [19] proposed an approach of integrating the strong motion data into the ambiguity-fixed precise point positioning. A tightly-integrated filter is developed to estimate coseismic displacements from raw GPS phase and pseudorange observations and raw strong motion data. In this filter, seismic data can improve the GPS estimates in terms of ambiguity fixing, besides, and the tightly-integrated filter can also provide displacements with better accuracy compared to the loosely-integrated approach [13,19].

In this study, we apply the tightly-coupled integration to analyze collocated GPS and seismic data collected during the 2011 Tohoku-Oki (Japan) and the 2010 El Mayor-Cucapah (Mexico) earthquakes. Time and frequency domain analysis show that the integrated displacement and velocity waveforms are more accurate than GPS-only or seismic-only results. The integrated displacement waveform can capture both transient phenomena (waves) and permanent or static deformation. From the integrated results, we detect the P-wave arrival, locate the epicenter, and extract the permanent offsets for static slip inversion and magnitude estimation.

## 2. Data Processing

For the strong motion station *r* at the epoch *k*, the accelerometer measurement *a_r_*,*_k_* can be expressed as:

(1) $$a_{r,k}={\tilde{a}}_{r,k}+b_{r,k}+\varepsilon_{a},\varepsilon_{a}∼N(0,Q_{a})$$

where, *ã_r_*,*_k_* is the true acceleration; *b_r_*,*_k_* is the acceleration bias which is estimated as a slowly time-varying parameter, *ε_a_* is the noise-like random error with variance *Q_a_*.

In loosely-integrated procedures, the GPS phase and pseudorange data are first analyzed to estimate station displacements by using either relative network positioning or precise point positioning. The GPS-derived displacement at the station *r* and epoch *k* can be expressed as:

(2) $$x_{r,k}={\tilde{x}}_{r,k}+\varepsilon_{x},\varepsilon_{x}∼N(0,Q_{x})$$

where, *ε_x_* ∼ *N*(0,*Q_x_*) is the GPS position noise.

The GPS displacement Equation (2) is combined with the accelerometer Equation (1) for the measurement update of the Kalman filter. The state vector *X_k_* can be expressed as:

(3) $$X_{r,k}=\left(\begin{array}{c} x_{r,k}\\ v_{r,k}\\ a_{r,k} \end{array}\right)$$

(4) $$\left(\begin{array}{c} x_{r,k}\\ v_{r,k}\\ a_{r,k} \end{array}\right)=\left(\begin{array}{ccc} 1 & \tau & \tau^{2}/2\\ 0 & 1 & \tau \\ 0 & 0 & 1 \end{array}\right)\left(\begin{array}{c} x_{r,k-1}\\ v_{r,k-1}\\ a_{r,k-1} \end{array}\right)$$

where, *x_r_*,*_k_* is the coordinate, *v_r_*,*_k_* is the velocity and *a_r_*,*_k_* is the acceleration; *τ* is the accelerometer sampling interval; the transition Equation (4) is used for time update of the Kalman filter.

The accelerometer data can be applied as strict constraints on the position variation between epochs and therefore improves GPS ambiguity resolution and outlier identification. Here, we apply the tightly-coupled integration Kalman filter to analyze raw GPS phase and pseudorange observations and raw strong motion data. The linearized equations for raw carrier phase and pseudo-range observations can be expressed as follows [20,21]:

(5) $$l_{r,j}^{s}=-u_{r}^{s}\cdot x_{r}+m_{r}^{s}\cdot Z_{r}-t^{s}+t_{r}+\lambda_{j}(b_{r,j}-b_{j}^{s})-I_{r,j}^{s}+\lambda_{j}N_{r,j}^{s}+\varepsilon_{r,j}^{s}$$

(6) $$p_{r,j}^{s}=-u_{r}^{s}\cdot x_{r}+m_{r}^{s}\cdot Z_{r}-t^{s}+t_{r}+c(d_{r,j}+{d_{j}}^{s})+I_{r,j}^{s}+e_{r,j}^{s}$$

where, $l_{r,j}^{s}$, $p_{r,j}^{s}$ denote “observed minus computed” phase and code observables from satellite *s* to receiver *r* at frequency *j*; $u_{r}^{s}$ is the unit direction vector from receiver to satellite; *x_r_* denotes the vector of the receiver position; *Z_r_* denotes tropospheric zenith wet delay; $m_{r}^{s}$ is the wet part of global mapping function; *t^s^* and *t_r_* are the clock errors of satellite and receiver respectively; *λ_j_* is the wavelength of the *j* frequency; *b_r_*,*_j_* is the receiver-dependent uncalibrated phase delay at the *j* frequency; $b_{j}^{s}$ is satellite-dependent uncalibrated phase delay; *d_r_*,*_j_* is the code bias of the receiver; $d_{j}^{s}$ is the code bias of satellite; $I_{r,j}^{s}$ is ionospheric delay on the path at the *j* frequency; $N_{r,j}^{s}$ is the integer phase ambiguity; $e_{r,j}^{s}$ is the pseudo-range measurement noise; $\varepsilon_{r,j}^{s}$ is measurement noise of carrier phase.

Integer ambiguity fixing in PPP requires not only precise satellite orbit and high-rate satellite clock corrections but also uncalibrated phase delay (UPD) [22]. With the received corrections of GPS satellite orbits, clocks and UPDs, the raw observation equations can be simplified as:

(7) $$l_{r,j}^{s}=-u_{r}^{s}\cdot x_{r}+m_{r}^{s}\cdot Z_{r}+t_{r}-\kappa_{j}\cdot I_{r,1}^{s}+\lambda_{j}N_{r,j}^{s}+\varepsilon_{r,j}^{s}$$

(8) $$p_{r,j}^{s}=-u_{r}^{s}\cdot x_{r}+m_{r}^{s}\cdot Z_{r}+t_{r}+\kappa_{j}\cdot I_{r,1}^{s}+e_{r,j}^{s}$$

At the epoch *k*, the state vector can be expressed as:,

(9) $$X_{r,k}={\left({x_{r,k}}^{T}{v_{r,k}}^{T}{a_{r,k}}^{T}Z_{r,k}t_{r,k}{I_{r,k}}^{T}{N_{r,k}}^{T}\right)}^{T}$$

The measurement update with raw GPS and accelerometer observations of Equations (1), (7) and (8) is applied at every GPS epoch. The time update of Equation (4) is performed for every accelerometer sample. The integer ambiguity resolution is attempted at every GPS epoch, L1 and L2 ambiguities are fixed simultaneously using integer estimation methods [23,24]. The ratio of the second minimum to the minimum quadratic form of residuals is applied to decide the correctness and confidence level of integer ambiguity candidate (the threshold for ratio test is set to 3 [25,26]).

## 3. Results and Discussion

The 2011 Mw 9.0 Tohoku-Oki earthquake (11 March 2011, 05:46:24 UTC) in Japan and the 2010 Mw 7.2 El Mayor-Cucapah earthquake (4 April 2010, 22:40:42 UTC) in Mexico were well recorded not only by strong motion stations, but also by high-rate GPS receivers. They are good examples to evaluate the performance of integrated displacements for which abundant high-rate GPS and strong motion records are available [1,27].

We firstly processed 1 Hz data of about 90 globally distributed real-time IGS stations using the EPOS-RT software of GFZ [28] in simulated real-time mode for providing GPS orbits, clocks and UPD corrections at 5 s sampling interval. Based on these corrections, we process the GPS and strong motion data collected at about thirty collocated stations during the Tohoku-Oki and El Mayor-Cucapah earthquakes. As PPP can be performed with a single GPS receiver, the integrated displacements are estimated on a pair-by-pair basis for each collocated GPS and strong motion pair.

For the 2011 Tohoku-Oki earthquake, the 1 Hz GPS data is collected at the GPS Earth Observation Network System (GEONET) stations operated by the Geospatial Information Authority (GSI) of Japan. One hundred Hz accelerometer data is collected from strong motion stations of the K-Net and Hi-Net. For the 2010 El Mayor-Cucapah earthquake, 5 Hz GPS data is collected from the California Real-Time Network (CRTN) and Plate Boundary Observatory (PBO). Two hundred Hz accelerometer data is collected from strong motion stations of the Southern California Seismic Network (SCSN) operated by the USGS (U.S. Geological Survey) and Caltech.

### 3.1. Comparison of GPS, Seismic and Integrated Waveforms

**Figure 1.**
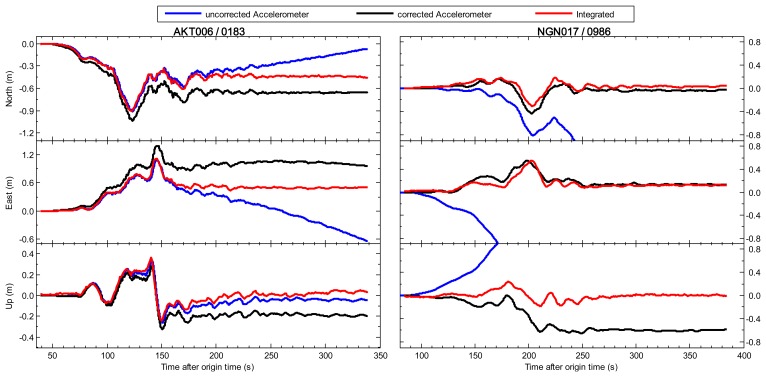
Comparison of uncorrected seismic-only and corrected seismic-only, and integrated displacements on the collocated AKT006 (seismic) and 0183 (GPS) pair and NGN017 (seismic) and 0986 (GPS) pair during the Tohoku-Oki earthquake on 11 March 2011. All sub-figures show the entire period of seismic shaking. The 100 Hz integrated displacement is shown by the red line. The 100 Hz seismic-only displacements without baseline correction and with baseline correction are respectively shown by the blue line and the black line, respectively.

We compare the integrated displacements with seismic-only waveforms obtained from double integration of raw acceleration data. The results of two collocated pairs AKT006/0183 and NGN017/0986 are shown in Figure 1 as an example. The left sub-figures show the entire period of the seismic shaking in north/east/up components at AKT006/0183, and the right ones show the seismic shaking at NGN017/0986 in the same three components. The GPS station 0183 (40.2154° N, 140.7873° E), which is located 251 km from the epicenter of Tohoku-Oki earthquake, is collocated with K-Net seismic station AKT006 (about 20 m away from GPS station), and the other pair NGN017 and 0986 station within 5 km distance, where the distance to the epicenter is about 480 km.

The uncorrected seismic displacements are traditionally observed from zero-order corrected with only consideration removing the pre-event mean bias. Although the dynamic motions can be determined, a linear or parabolic drift is apparent in the latter part of each displacement time series, and the permanent coseismic offset is lost in a seismic-only solution. The corrected seismic displacements are derived from the baseline-corrected strong motion recordings which are processed using the automatic empirical baseline correction scheme proposed by Wang [29]. Although the corrected seismic displacements have a high degree of similarity of the dynamic component with the integrated results, they still maintain several decimeter differences in permanent coseismic offsets due to the effect of the residual baseline bias error. From the integrated displacement waveforms, there are obvious permanent coseismic offsets which are about 0.47 m, 0.51 m, and 0.03 m in the north, east, and up components at station AKT006/0183, while the permanent offsets of station NGN017/0986 are relatively small, about 0.04 m in the north, 0.12 m in the east, and 0.01 m in the up components. It is demonstrated that the Wang's method is currently considered to be the most robust seismic-alone one [30], but more accurate displacements without offsets should be relied on the GPS-aided baseline correction method [11].

**Figure 2.**
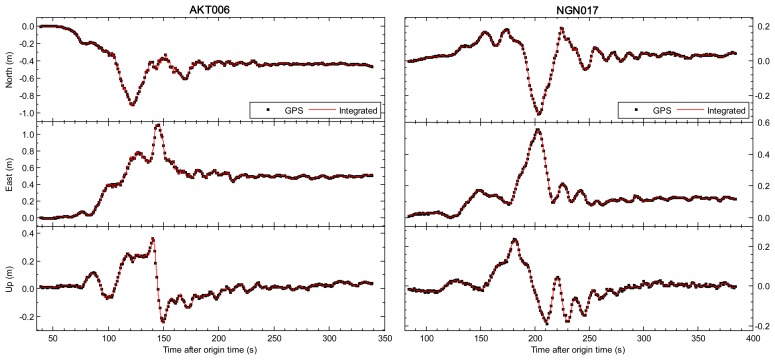
Comparison of GPS-only and tightly-integrated displacements on the collocated AKT006 (seismic) and 0183 (GPS) pair and NGN017 (seismic) and 0986 (GPS) pair during the Tohoku-oki earthquake on 11 March 2011. The sub-figures show from top to bottom the entire period of seismic shaking in north, east and up components respectively. The 1 Hz GPS-only and 100 Hz tightly-integrated displacements are shown respectively by the black crosses and the red line.

**Figure 3.**
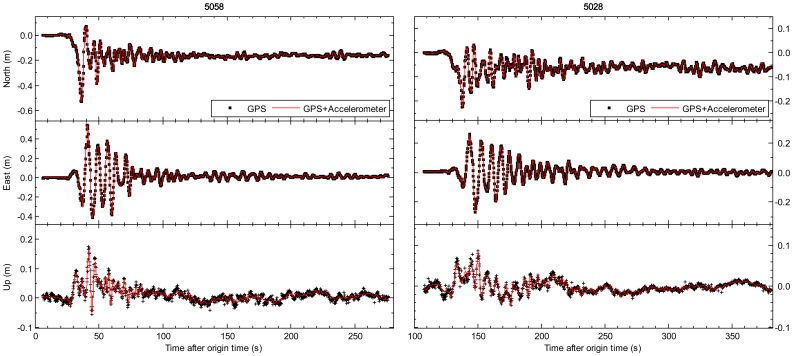
Comparison of GPS-only and tightly-integrated displacements on the collocated 5058 (seismic) and P496 (GPS) pair and 5028 (seismic) and P744 (GPS) pair during the El Mayor–Cucapah earthquake on 4 April 2010. The sub-figures from top to bottom show the entire period of seismic shaking in north, east and up components respectively. The 5 Hz GPS-only and 200 Hz tightly-integrated displacements are shown respectively by the black cross symbols and red lines.

In Figures 2 and 3, we compare the tightly-integrated displacements (the red line) and GPS-only displacements (the black cross symbols). The results of the AKT006/0183 and NGN017/0986 pairs are respectively shown in the left and right side of Figure 2, and the similar results of the 5058/P496 and 5028/P744 pairs are also shown in Figure 3. The GPS station P496, which is located about 60 km from the epicenter of 2010 El Mayor-Cucapah earthquake, is collocated with SCSN seismic station 5058 (about 70 m separation). The other pair P744 and 5028 station are within 140 m of each other, and the distance from them to the epicenter is about 65 km. All sub-figures from top to bottom depict the entire period of seismic shaking in north, east and up components. We can see that the integrated displacements are in good agreement with GPS-only solution in terms of peak displacements, permanent offsets and long-period stability. However, it is clearly shown that the GPS-only displacements are with lower sampling rate and higher noise compared to the integrated displacements. The root mean square (RMS) values of GPS-only solution (10 min pre-event displacement series) are 1.1, 1.1 and 3.0 cm respectively in north, east and vertical components. The precision of integrated displacement is significantly improved by precise dynamical information provided by seismic sensors.

**Figure 4.**
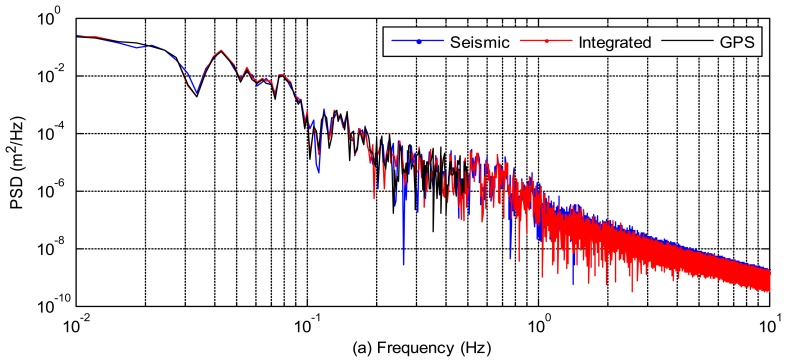

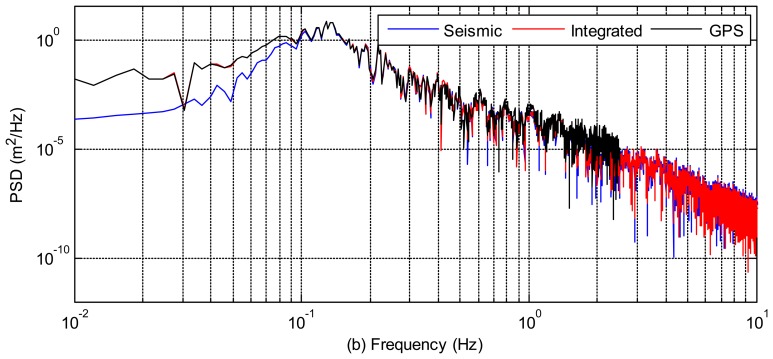
Power spectral densities. (**a**) Power spectral density for 1 Hz GPS displacements at station 0183 (the black line), 100 Hz seismic displacements at AKT006 (the blue), and 100 Hz tightly-integrated displacement waveforms at AKT006/0183 (the red line). (**b**) Power spectral density for 5 Hz GPS displacements at P744 (the black line), 200 Hz seismic displacements at 5028 (the blue line), and 200 Hz tightly-integrated displacements at P744/5028 (the red line).

The power spectral densities of the three kinds of displacements (GPS-only, seismic-only, and integrated displacements) at AKT006/0183 and P744/5028 pairs are also compared in Figure 4 to illustrate the frequency content of the signal. The frequency domain analysis of these waveforms shows in which frequency bands each data type is reliable. GPS performs better at lower frequencies and seismic sensor is better at higher frequencies. We can see that the power spectral densities of integrated displacements follow the GPS-only spectrum at the low frequencies and the seismic-only spectrum at the high frequencies. From the power spectral density analysis, we can also infer that the integrated waveform is more precise and accurate than the GPS-only or seismic-only waveforms. An accurate broadband waveform, which has the advantages of both sensors, has been achieved.

### 3.2. Detection of P-Wave Arrival

**Figure 5.**
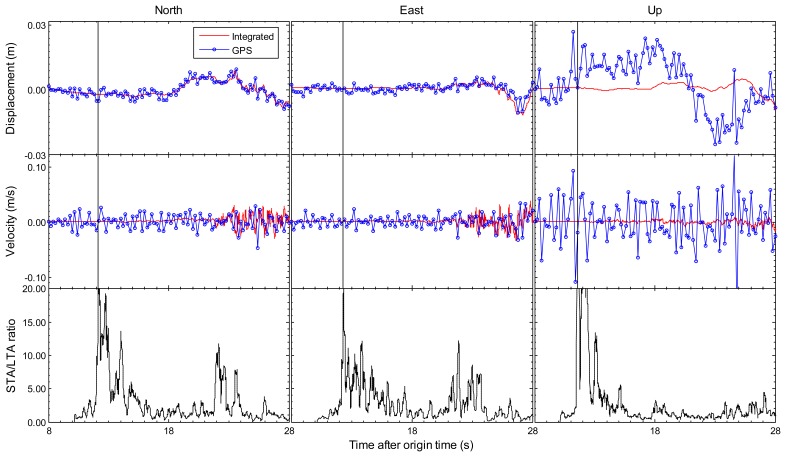
An enlarged view of the first 20 s of the coseismic displacements and velocities in all three components on the collocated 5028 (seismic) and P744 (GPS) pair during the El Mayor–Cucapah earthquake. The 5 Hz GPS-only and 200 Hz tightly-integrated displacements and velocities are respectively shown by the blue dotted lines and red lines. The bottom sub-figures are STA/LTA ratio results based on tightly-integrated results, which show the first arrival time of seismic wave. The sub-figures show, from left to right, the north, east and up components.

**Figure 6.**
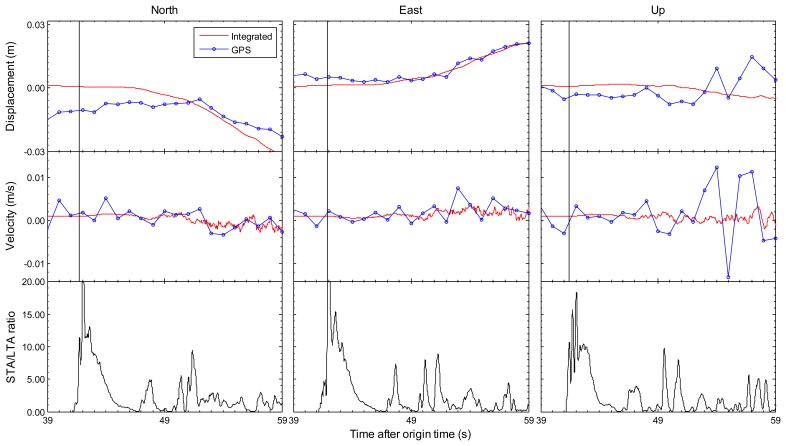
An enlarged view of the first 20 s of the coseismic displacements and velocities in all three components on the collocated AKT006 (seismic) and 0183 (GPS) stations during the Tohoku-oki earthquake on 11 March 2011. The 1 Hz GPS-only and 100 Hz tightly-integrated displacements and velocities are respectively shown by the blue dotted lines and red lines. The bottom sub-figures are STA/LTA ratio results based on tightly-integrated results, which show the first arrival time of seismic wave. The sub-figures show, from left to right, the north, east and up components.

Earthquake monitoring and early warning systems not only depend on the accurate estimation of permanent displacements, but also rely on the capability of the sense of P-wave arrival which is employed to predict the arrival and intensity of destructive S and surface waves. Figures 2 and 3 have shown that the integrated results could get accurate permanent offsets. The following sections mainly focus on another issue. The enlarged view of the first 20 s of the integrated and GPS-only results for station 5028/P744 is shown in Figure 5, and the similar enlarged view for station AKT006/0183 is shown in Figure 6. From coseismic displacement and velocity waveforms, we can observe that the GPS-only solution is noisy and has a precision limited to several millimeters in displacement and few centimeters per second in velocity. The vertical component is much noisier as expected, due to the satellite constellation configuration and the high correlation between zenith tropospheric delay and the height component. The precision of vertical displacement is of the order of few centimeters, and vertical velocity precision is around several centimeters per second, which is not enough to detect P-wave accurately. With the aid of the seismic data, the tightly-integrated filter is capable of producing a precise integrated displacement and velocity waveform, especially in the up component. The small-amplitude P-wave can be clearly observed, and the P-wave arrival can also be easily detected from the integrated waveform. This is a significant improvement over the GPS-only solution where earthquake signal is detected only after the S-wave arrival, which is generally a few seconds later than the P-wave arrival for near-field stations.

The bottom sub-figures in Figures 5 and 6 are STA/LTA ratio values based on tightly-integrated results for north/east/up components, which are used to pick up the earthquake P-wave arrival. The short-term average (STA) through long-term average (LTA) picker is the most broadly used automatic algorithm in seismology [31]. It continuously calculates the average values of the absolute amplitude of a seismic signal in two consecutive moving-time windows. The short time window (STA) is sensitive to seismic events while the long time window (LTA) provides information about the temporal amplitude of seismic noise at the site [32]. When the ratio of both exceeds a pre-set threshold means the arrival of P-wave. The STA/LTA picker parameter settings are always a tradeoff between several seismological and instrumental considerations. For these two earthquake events in this paper, the STA window duration is 0.2 s, the LTA window duration is 2 s, and the pre-set threshold is set to 10. We can clearly identify P-wave arrivals in the STA/LTA ratio time series. It is noted that the P-wave appears in vertical component first and in the horizontal components a few milliseconds later. The detected earthquake P-wave arrival time of station AKT006/0183 is 41.41 s compared with the USGS reference value 41.55 s calculated by TauP Toolkit [33], and the P-wave arrival time of station 5028/P744 is 11.49 s compared with the reference value 11.58 s. It is demonstrated that the integrated results could be used to pick up an accurate P-wave arrival time. However, it is difficult for the GPS-only solution to be accurately identified P-waves because of the significantly less precision. Thus, the integrated result improves on both seismic-only and GPS-only methods, by providing the full spectrum of seismic motions from the detection of P-wave arrivals to the estimation of permanent offsets.

When P-waves are detected at four or more near-field GPS/strong motion pairs, the epicenter, the velocity of earthquake wave and the origin time can be determined by using a least squares method as follows:

(10) $$\{\begin{array}{l} (\frac{x_{1}-x_{0}}{d_{1}^{0}}-\frac{x_{2}-x_{0}}{d_{2}^{0}})\cdot dx+(\frac{y_{1}-y_{0}}{d_{1}^{0}}-\frac{y_{2}-y_{0}}{d_{2}^{0}})\cdot dy+(t_{1}-t_{2})\cdot v-(d_{1}^{0}-d_{2}^{0})=0\\ (\frac{x_{1}-x_{0}}{d_{1}^{0}}-\frac{x_{3}-x_{0}}{d_{3}^{0}})\cdot dx+(\frac{y_{1}-y_{0}}{d_{1}^{0}}-\frac{y_{3}-y_{0}}{d_{3}^{0}})\cdot dy+(t_{1}-t_{3})\cdot v-(d_{1}^{0}-d_{3}^{0})=0\\ \vdots \\ (\frac{x_{1}-x_{0}}{d_{1}^{0}}-\frac{x_{n}-x_{0}}{d_{n}^{0}})\cdot dx+(\frac{y_{1}-y_{0}}{d_{1}^{0}}-\frac{y_{n}-y_{0}}{d_{n}^{0}})\cdot dy+(t_{1}-t_{n})\cdot v-(d_{1}^{0}-d_{n}^{0})=0\\ t_{0}=\frac{\sum_{i=1}^{n}\left(t_{i}-\frac{d_{i}}{v}\right)}{n} \end{array}$$

where, *x*_0_,*y*_0_ denote the approximate coordinates of the epicenter; *x_i_*,*y_i_*(*i* = 1⋯*n*)denote the coordinates of the *i*th station; $d_{i}^{0}$ denotes the distance from the *i*th station to the approximate coordinates of epicenter; *dx*,*dy* denote the increments of epicenter; *v* denotes velocity of earthquake wave; *t_i_* denotes the arrival time of earthquake wave at the *i*th station; *d_i_* denotes the distance from the *i*th station to the epicenter; *t*_0_ denotes the origin time. Several iterations are required to avoid the linearization error.

In order to test this technique, the five GPS/strong motion pairs where P-wave is detected earliest during the El Mayor-Cucapah earthquake are used. The detected earthquake P-wave arrival time is 0.09 s, 0.15 s, 0.11 s, 0.10 s, and 0.13 s later than the USGS reference values of P-wave arrival time at the five pairs. The epicenter estimate is roughly 2.5 km away from the U.S. Geological Survey (USGS) epicenter estimate. The origin time estimate is 0.12 s later than the USGS reference value of 22:40:57 (GPS time). The accurate detection of P-wave arrival is critical for earthquake early warning, as it allows for prediction of the arrival of the destructive S-wave. The P-wave-based earthquake parameters such as epicenter and origin time can be released before the S-wave arrival.

### 3.3. Extraction of Permanent Offset and Fault Slip Inversion

**Figure 7.**
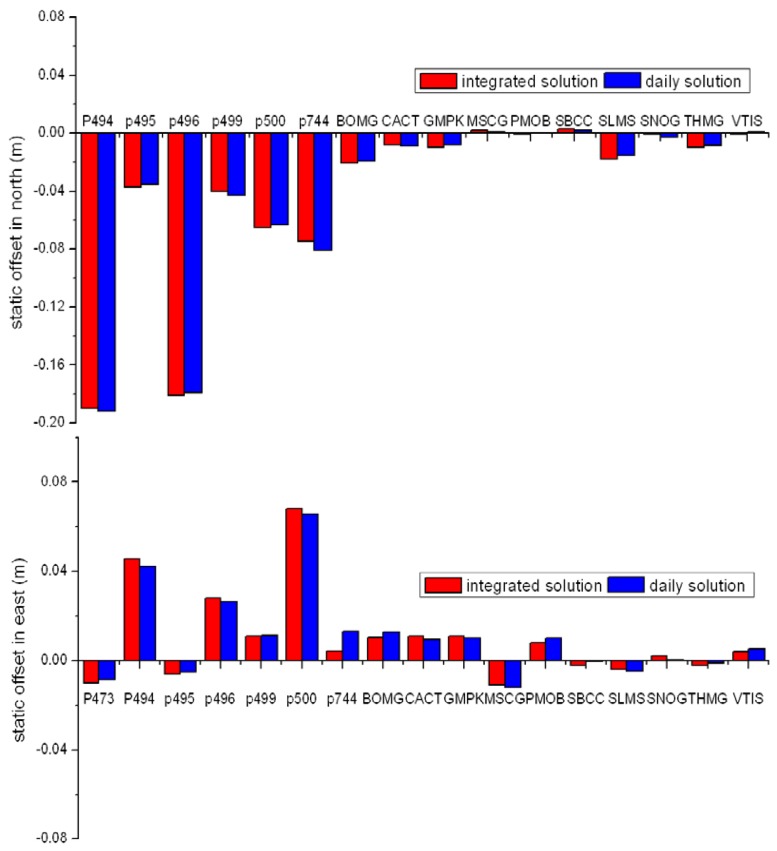
Comparison of the permanent (static) offsets from the tightly-integrated solution and the post-processed daily solution. The blue rectangle shows static offsets derived from the static PPP solution with daily observations (the difference between daily solutions of the day before the earthquake and the day after the earthquake). The red rectangles show the static offsets derived from the real-time tightly-integrated solution.

In addition to P-wave arrival time, the important information, provided by the integrated position series, is the permanent offset. We use the real-time algorithm proposed by Allen and Ziv [1] to remove dynamic oscillations and extract these offsets. The permanent offsets derived from integrated solution (about 1 min after the arrival of the earthquake wave) are compared with the ones from the post-processed daily solution in Figure 7. The RMS of the differences between them is about 3.7 mm.

We derived the spatial distribution of the fault slip using the coseismic displacements obtained from both the real-time tightly-integrated solution and the post-processed daily solution. In the same way as done by Li *et al.* [34], the fault geometric parameters (strike 312°/dip 88°) are adopted from the Global Centroid Moment Tensor (GCMT) solution of the earthquake. The rake angle (slip direction relative to the strike) is allowed to vary ±20° around the GCMT solution of 186°. The fault size is given to be 130 km along the strike and 20 km along the dip, which is then divided into 26 × 4 = 104 sub-faults. In the inversion, the data is weighted twice as much for the two horizontal components as for the vertical component.

**Figure 8.**
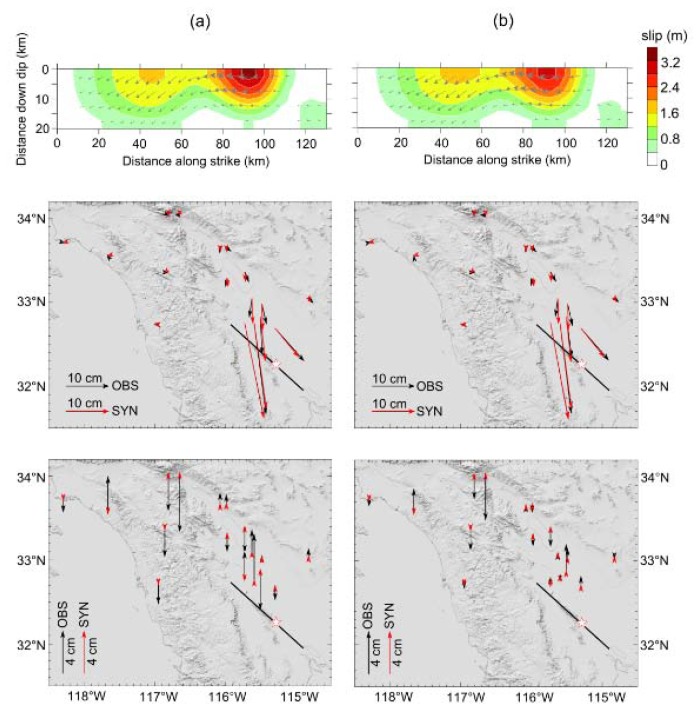
Fault slip inversion. (**a**) Inversion with permanent coseismic displacements obtained from real-time tightly-integrated solution; (**b**) Inversion with post-processed daily solution. From top to bottom are the inverted fault slip distributions, comparisons between the observed and the synthetic displacements on the horizontal components, and on the vertical components, respectively.

The inversion results are shown in Figure 8. The two inversions result in scalar seismic moments of 7.27 × 10^19^ Nm and 7.18 × 10^19^ Nm respectively, equivalent to moment magnitude of Mw7.18 for both. Although there are some differences existing on the maximum slip values which may be caused by the inconsistency in the vertical component between the two datasets, the two inversion results are quite similar not only in the moment magnitude, but also in the slip distribution pattern. The major slip area occurred at a very shallow depth (near the surface) at about 90 km along the strike direction on the fault plane. The rake variation shows that there is a purely right lateral strike slip at the northwest of the fault, and a minor normal fault component occurs at the south east of the fault. Considering the hypocentral location, we can confirm that this earthquake is an asymmetric bilateral rupture event: the rupture mainly propagates northwestward from the hypocenter during the source process. Overall, the comparison of the two inversion results shows that the integrated solution can provide a reliable estimation of earthquake magnitude and even of the fault slip distribution in real time.

## 4. Conclusions

We analyzed the collocated GPS and seismic data collected during the 2011 Tohoku-Oki (Japan) and the 2010 El Mayor-Cucapah (Mexico) earthquakes using a tightly-coupled integration. The integrated waveform takes the advantages of both sensors and is more precise and accurate than the GPS-only or seismic-only waveforms. The power spectral densities of integrated displacements follow the GPS-only spectrum at the low frequencies and the seismic-only spectrum at the high frequencies.

The integrated displacements can provide the full spectrum of the seismic motion allowing the detection of P-wave arrivals and the estimation of permanent offsets. Small-scale features including P-waves are visible in the integrated displacement and velocity waveforms. The P-wave arrival can be picked up accurately and used for reliable determination of epicenter and origin time. Permanent offsets can also be extracted with high accuracy and used for reliable fault slip inversion and magnitude estimation. These earthquake parameters are critical for earthquake/tsunami monitoring and early warning systems.
